# Soluble urokinase receptor released from human carcinoma cells: a plasma parameter for xenograft tumour studies

**DOI:** 10.1038/sj.bjc.6690678

**Published:** 1999-09

**Authors:** C Holst-Hansen, M J A G Hamers, B E Johannessen, N Brünner, R W Stephens

**Affiliations:** The Finsen Laboratory, Rigshospitalet, Strandboulevarden 49 Bld 86.2, Copenhagen Ø, DK-2100, Denmark

**Keywords:** uPAR, soluble receptor, human cancer xenografts

## Abstract

The urokinase plasminogen activator receptor (uPAR) plays a critical role in urokinase-mediated plasminogen activation and thereby in the process leading to invasion and metastasis. Soluble urokinase receptor (suPAR) is released from tumours, and in cancer patients the blood level of soluble receptor is increased. Using an enzyme-linked, immunosorbent assay (ELISA)-specific for the human urokinase receptor, release of soluble receptor was measured in cultures of human breast carcinoma cells, in tumour extracts and in plasma from mice with xenografted human tumours. Soluble human urokinase receptor (shuPAR) was released into culture supernatant during the growth of the human breast cancer cell line MDA-MB-231 BAG, and the level of shuPAR in conditioned medium determined by ELISA was a linear function of both viable cell number and time of incubation. Western blotting showed that the form of shuPAR measured by ELISA in conditioned medium consisted virtually exclusively of the three-domain full-length protein, while uPAR in cell lysates consisted of full-length uPAR as well as the domains (2+3) cleavage product. shuPAR was also released into the plasma of nude mice during growth of MDA-MB-231 BAG, MDA-MB-435 BAG and HCT 116 cells as subcutaneously xenografted tumours. Western blotting demonstrated that the shuPAR released from the xenografted human tumours into plasma consisted of the three-domain full-length protein, despite the finding of some cleaved uPAR in detergent extracts of tumour tissue. The levels of shuPAR determined by ELISA in the plasma of host mice during the growth of xenografted cell lines were highly correlated with tumour volume. © 1999 Cancer Research Campaign


*British Journal of Cancer* (1999) **81**(2), 203–211
© 1999 Cancer Research Campaign
Article no. bjoc.1999.0678

### Soluble urokinase receptor released from human carcinoma cells: a plasma parameter for xenograft tumour studies

**C Holst-Hansen[1], MJAG Hamers, BE Johannessen, N Brünner and RW Stephens**

The Finsen Laboratory, Rigshospitalet, Strandboulevarden 49, Bld 86.2, DK-2100 Copenhagen Ø, Denmark

**Summary** The urokinase plasminogen activator receptor (uPAR) plays a critical role in urokinase-mediated plasminogen activation and thereby in the process leading to invasion and metastasis. Soluble urokinase receptor (suPAR) is released from tumours, and in cancer patients the blood level of soluble receptor is increased. Using an enzyme-linked, immunosorbent assay (ELISA)-specific for the human urokinase receptor, release of soluble receptor was measured in cultures of human breast carcinoma cells, in tumour extracts and in plasma from mice with xenografted human tumours. Soluble human urokinase receptor (shuPAR) was released into culture supernatant during the growth of the human breast cancer cell line MDA-MB-231 BAG, and the level of shuPAR in conditioned medium determined by ELISA was a linear function of both viable cell number and time of incubation. Western blotting showed that the form of shuPAR measured by ELISA in conditioned medium consisted virtually exclusively of the three-domain full-length protein, while uPAR in cell lysates consisted of full-length uPAR as well as the domains (2+3) cleavage product. shuPAR was also released into the plasma of nude mice during growth of MDA-MB-231 BAG, MDA-MB-435 BAG and HCT 116 cells as subcutaneously xenografted tumours. Western blotting demonstrated that the shuPAR released from the xenografted human tumours into plasma consisted of the three-domain full-length protein, despite the finding of some cleaved uPAR in detergent extracts of tumour tissue. The levels of shuPAR determined by ELISA in the plasma of host mice during the growth of xenografted cell lines were highly correlated with tumour volume.

© 1999 Cancer Research Campaign

**Keywords:** uPAR; soluble receptor; human cancer xenografts

It is now well established that proteolytic enzymes produced by cancer cells and/or cells in tumour stroma are involved in the intensive tissue remodelling, which accompanies cancer cell invasion and metastasis (Danø et al, 1994; Andreasen et al, 1997). Among the several enzyme systems expressed in cancer tissue, urokinase-catalysed plasminogen activation is thought to play a key role in tissue degradation (Danø et al, 1985; Shapiro et al, 1996), activation of pro-metalloproteinases (He et al, 1989; Baramova et al, 1997) and activation of cytokines (Lyons et al, 1990), all of which could lead to increased metastatic potential of the cancer cells. The urokinase plasminogen activator (uPA) has a specific cell surface receptor (uPAR) that is a 55–60 kDa, highly glycosylated protein, consisting of three homologous domains, the amino-terminal domain 1 as well as the other two domains being important for high affinity binding of the growth factor domain of uPA (Ploug et al, 1998). The carboxy-terminal of uPAR is attached to the cell surface by a glycosylphosphatidylinositol (GPI)-anchor, so that binding of the inactive proenzyme of uPA to uPAR and concomitant cell surface binding of plasminogen strongly enhances plasmin generation (Ellis et al, 1991). Binding of uPA to uPAR also promotes binding of uPAR to the adhesive matrix protein vitronectin, a process which is reversed by the plasminogen activator inhibitor type-1 (PAI-1), a competitor of uPAR binding to vitronectin (Deng et al, 1996).

In vitro studies have suggested that full-length three-domain uPAR protein can be released from the surface of human cells (Lau and Kim, 1994), but the mechanism of this is unclear. Bacterial phospholipase C can cleave the glycolipid anchor (Ploug et al, 1991), while no mammalian phospholipase of appropriate specificity is known. Proteolytic cleavage between domains 1 and 2 can release soluble domain 1, but no free domain 1 has been demonstrated in cell culture supernatants so far. If cleavage by phospholipases and/or proteases occurred in vivo, soluble uPAR (suPAR) consisting of the full-length three-domain protein, or the domains (2+3 and 1) form of the receptor, all without glycolipid anchor, could be expected in peripheral blood. Recently we have reported that shuPAR is found in sera and plasma from healthy blood donors at a median concentration of approximately 1.2 ng ml$^{-1}$ (Stephens et al, 1997), but the mechanism of release remains unclear. uPAR is known to be displayed on the surface of human blood monocytes and neutrophils (Plesner et al, 1997), so that senescence of these cells could be one source of suPAR in blood. The differentiation of megakaryocytes and formation of platelets in bone marrow may also contribute significant levels of suPAR to the blood compartment (Wohn et al, 1997).

In recent studies of patients with non-small cell lung cancer (Pappot et al, 1997), breast and colon cancer (Stephens et al, 1997) and ovarian cancer (Sier et al, 1998), the concentration of shuPAR was found to be significantly increased in patient plasma as

*Received 23 November 1998*
*Revised 17 March 1999*
*Accepted 24 March 1999*

*Correspondence to:* The Finsen Laboratory, Rigshospitalet, Strandboulevarden 49, Bld 86.2, DK-2100 Copenhagen Ø, Denmark compared to healthy individuals. We proposed that the increase in blood shuPAR in cancer patients was due to release from the surface of tumour and/or stromal cells in the cancer tissue (Pappot et al, 1997; Stephens et al, 1997). In patients with ovarian cancer, at least part of the suPAR present in the ascites fluid consists of all three protein domains, and thus retains its ability to bind uPA (Pedersen et al, 1993). However, no direct demonstration has been reported of the relationship between plasma suPAR levels and tumour burden, nor has the possible biological role of suPAR been clarified.

Previous studies have shown that uPAR as measured in extracts of tumour tissue provides significant prognostic information. High levels of uPAR in extracts of tumour tissue is associated with short survival in patients with lung, colon and breast cancers (Ganesh et al, 1994; Pedersen et al, 1994; Grøndahl-Hansen et al, 1995). These prognostic studies have now been extended to measurements of shuPAR in peripheral blood, and we have recently shown that high levels of shuPAR in plasma are associated with shorter survival in patients with colorectal cancer (Stephens et al, 1999) and ovarian cancer (Sier et al, 1998). While elevated levels of shuPAR were found even in early-stage disease (e.g. Dukes' B colon cancer), higher levels of shuPAR were associated with advanced disease, suggesting that plasma shuPAR levels may bear a complex relation to both tumour invasive activity as well as tumour burden.

Using a newly developed sensitive and specific enzyme-linked immunosorbent assay (ELISA) for human uPAR (Stephens et al, 1997), together with immunoabsorption and Western blotting, we have now measured and characterized the shuPAR released in vitro from human carcinoma cells. Moreover, we have quantitated and characterized shuPAR found in the host plasma of nude mice carrying human cancer xenografts. The experimental data demonstrate that plasma levels of shuPAR reflect tumour burden in animal models.

### MATERIALS AND METHODS

### Antibodies and uPAR standards

A mouse monoclonal antibody (mAb) designated clone R2, with high affinity and specificity for domain 3 of human uPAR was used as capture antibody for the shuPAR ELISA (Rønne et al, 1995), and purified rabbit polyclonal anti-human uPAR antibodies (Stephens et al, 1997) were used for detection in both ELISA and Western blotting. The mouse mAbs against human uPAR designated clones R2 and R4 (Rønne et al, 1995) were used for immunoabsorption, which also employed a control mouse mAb against the 2,4,6-trinitrophenyl hapten (TNP) (Rønne et al, 1995).

Purified recombinant shuPAR (Rønne et al, 1994) was used as standard in the ELISA. Full-length shuPAR and chymotrypsin-cleaved shuPAR for use as standards in the Western blotting were prepared as described (Ploug et al, 1994). Purified recombinant soluble mouse uPAR (smuPAR) from a Chinese hamster ovary cell expression system was a kind gift from Dr Helene Solberg.

### Cell lines

The MDA-MB-231 human breast cancer cell line was originally obtained from ATCC (Maryland, USA) and the MDA-MB 435 human breast cancer cell line was kindly provided by Dr Janet Price (MD Anderson Hospital, Houston, TX, USA). The breast cancer cell lines were transduced with the BAG vector as described (Brünner et al, 1992). We have previously characterized these two cell lines with regard to protein expression of the uPA system in vitro (Holst-Hansen et al, 1996). The cells were routinely propagated in Eagle's modified essential medium (MEM) with Glutamax-1 (Life Technologies) and supplemented with 5% fetal calf serum (FCS). The HCT 116 human colon cancer cell line was kindly provided by Dr A Dragone (Boehringer Mannheim, Monza, Italy). The cells were routinely propagated in McCoy's 5A with Glutamax-1 (Life Technologies), 20 mM HEPES and 10% FCS. All cell lines were tested by immunofluorescence, polymerase chain reaction (PCR) and DNA staining and found to be free from mycoplasma contamination.

### Conditioned media

Cells were harvested with trypsin–EDTA and added in complete FCS supplemented medium to 24-well cluster plates at a cell density of 25 000 cells per well or as indicated. Next day the adherent monolayers were washed twice in phosphate-buffered saline (PBS) and the medium was changed to serum-free conditions using 0.1% bovine serum albumin (BSA; Fraction V, Sigma) and 10 mM HEPES in Eagles MEM supplemented with Glutamax-1 (SFM). Cells were incubated at 37°C for 24 or 48 h as indicated. Conditioned media were collected and centrifuged at 10 000 ***g*** for 5 min at 4°C. Some conditioned media were ultracentrifuged at 105 000 ***g*** for 60 min at 4°C. Supernatants were frozen and stored at –80°C until analysed. Viable cells were quantitated by the 3-(4,5-dimethylthiazal-2-y1)-2,5-diphenyltetrazolium bromide (MTT)-method (Sigma Chemicals) as described previously (Holst-Hansen et al, 1996).

### Cell lysates

For cell lysates, MDA-MB-231 BAG cells were cultured under serum-free conditions for 48 h and then harvested by scraping with a rubber policeman. Cells were lysed by addition of 1 ml of lysis buffer (0.1 M Tris–HCl, pH 8.1, 1% Triton X-114, 10 mM EDTA, 10 μg $ml^{-1}$ aprotinin, 1 mM phenylmethylsulphonyl fluoride (PMSF)) to $5 \times 10^7$ cells. The lysates were subjected to temperature-induced phase separation (Behrendt et al, 1990), and the resulting supernatants were subsequently analysed by Western blotting (see below).

### shuPAR ELISA

shuPAR was determined in undiluted culture supernatants and in 1:10 dilutions of mouse plasma made in a sample dilution buffer of 50 M phosphate, pH 7.2, 0.1 M sodium chloride, 10 mg $ml^{-1}$ BSA (Fraction V, Boehringer Mannheim), 1 mg $ml^{-1}$ Tween-20, using a new kinetic ELISA which meets strict criteria of specificity and sensitivity (Stephens et al, 1997). To avoid a high background signal from mouse plasma, the catching and detection layers were inverted, so that the catching layer consisted of the monoclonal antibody R2 (coated at 2 μg $ml^{-1}$), which is directed against domain 3 of the human uPAR molecule. Thus both the full-length (domains 1+2+3) and proteolytically cleaved (domains (2+3)) forms of shuPAR were measured by this assay. The detection layer consisted of rabbit polyclonal antibodies to human uPAR (used at 1 μg $ml^{-1}$), as previously used for capturing shuPAR in ELISA (Stephens et al, 1997). A monoclonal anti-rabbit immunoglobulin–alkaline phosphatase conjugate (Sigma Chemicals, St Louis, MO, USA; dilution 1:2000) was used in the final step, so that rate

 © 1999 Cancer Research Campaign measurements could be automatically collected over a 1 h incubation period in a Ceres 900™ plate reader (Bio-Tek Instruments, Winooski, VT, USA). The limit of detection for the assay was 0.003 ng $ml^{-1}$. The intra-assay variation for a pool of human citrate plasma was 4.8% ($n$ = 21), and the inter-assay variation for 30 successive assays of aliquots of the same plasma pool (on different days) was 7.6%. shuPAR was evidently stable in frozen plasma for at least several months.

### Recovery of standard shuPAR and smuPAR signal in mouse plasma

The recovery of signal from standard shuPAR was tested after addition to a 1:10 dilution of mouse citrate plasma pool. Standard shuPAR was added to the diluted plasma to give final concentrations in the range 0–1.0 ng shuPAR $ml^{-1}$. The recoveries in each case were calculated from the slopes of the lines representing shuPAR signal as a function of concentration, where 100% recovery was defined as the slope obtained when shuPAR was diluted in the sample dilution buffer (see ELISA above).

The recovery of signal from standard smuPAR was also tested after addition to a 1:10 dilution of mouse citrate plasma pool. Standard smuPAR was added to the diluted plasma to give final concentrations in the range 0–10 ng smuPAR $ml^{-1}$. One hundred per cent recovery was defined as the slope obtained when smuPAR was diluted in the sample dilution buffer.

### Animal experiments

Six- to 8-week-old intact female *nu/nu*-META/Bom (Bomholtgaard, Ry, Denmark) mice were used in all experiments. The mice were maintained according to the regulations set forth by the Danish Laboratory Animal Ethical Committee. For all tumour experiments, the cells for implantation were harvested from subconfluent (70–80%) monolayer cultures by scraping with a rubber policeman. The cell suspension ($10 \times 10^6$ cells $ml^{-1}$ in PBS) was inoculated ($2 \times 10^6$ cells per site) subcutaneously and bilaterally into the abdominal flanks of the recipient mice. The growth of tumours was monitored by measurement every 2–3 days of perpendicular diameters. Tumour volumes were estimated by calculation using the formula 0.5 ($width^2 \times length$).

Blood was collected from anaesthetized mice by cardiac puncture and rapidly mixed with cold trisodium citrate (0.1 vol) to give a final concentration of 12.9 mM citrate. The plasma was separated from blood cells within 1 h by centrifugation at 2000 ***g*** for 30 min at 4°C, and the supernatant was stored frozen at –80°C prior to assay.

Xenograft tumours were surgically removed, cut into pieces and immediately frozen in liquid nitrogen and stored at –80°C until use. Frozen tumours were pulverized with a precooled powder pistol. Tumour powder was suspended (100 mg tumour powder to 1 ml of lysis buffer) in lysis buffer (0.1 M Tris–HCl, pH 8.1, 1% Triton X-114, 10 mM EDTA, 10 µg $ml^{-1}$ aprotinin, 1 mM PMSF). The extracts were assayed by ELISA for uPAR antigen level. In addition, the extracts were subjected to temperature-induced phase separation (Behrendt et al, 1990), and the resulting supernatants were subsequently analysed by Western blotting (see below). Protein concentration of the supernatants was determined using the Bio-Rad protein assay (Bio-Rad, Richmond, CA, USA) with BSA (Fraction V, Sigma) as standard.

### Northern blotting

Total RNA was isolated from primary tumours established in the nude mice from the two breast cancer cell lines, using the acid guanidine–thiocyanate–phenol–chloroform method (Chomczynski and Sacchi, 1987). The total amount of RNA isolated was determined from the absorption at OD260, assuming that 1 OD260 unit is equivalent to 40 µg RNA.

The following DNA probes were used: p-uPAR-1, a plasmid carrying the entire human uPAR cDNA (Roldan et al, 1990); pHFβA-3′UT, coding for the 3′-untranslated region of human β-actin cDNA (Ponte et al, 1984). The probes were labelled by the random primer method (Feinberg and Vogelstein, 1983), using dCTP 5′-[α-$^{32}$P]triphosphate.

For Northern blots 15 µg of total RNA from each sample were size-fractionated by electrophoresis in 1.0% denaturating agarose gels containing formamide and transferred to reinforced nitrocellulose membranes (Rømer et al, 1994). Hybridization of blots was carried out at 42°C for 16 h in 50% formamide, 10 × Denhardt's solution, 0.5% sodium dodecyl sulphate (SDS), 5 × sodium–saline citrate (SSC), 200 µg $ml^{-1}$ salmon sperm DNA, 10% dextran sulphate, 200 µg $ml^{-1}$ yeast–tRNA and $2 \times 10^9$ dpm $\mu g^{-1}$ of $^{32}$P-labelled probes. The filters were washed at 65°C for 1 h in 2 × SSC, 0.1% SDS and at 65°C for 1 h in 0.2 × SSC, 0.5% SDS and at 65°C for 30 min in 0.1 × SSC, 0.5% SDS. Autoradiography was performed at –80°C using Kodak T-MAT-G films and Kodak Lanex X-Omatic intensifying screens.

### Immunoabsorption and Western blotting

Mouse citrate plasma was diluted 1:10 in sample dilution buffer (see ELISA above) to a final volume of 2 ml. The diluted sample was recycled five times through a 2.0 ml column of protein A-Sepharose (Pharmacia Biotech) in order to remove mouse IgG. The run-through was then applied to and recycled five times through a 1.0 ml column of protein A-Sepharose bearing the R2 monoclonal antibody to human uPAR, followed by extensive washing. After plugging of the column, 200 µl of Laemmli Sample Buffer™ (Bio-Rad) was applied and the bound proteins were eluted and heated to 90°C for 5 min. SDS-polyacrylamide gel electrophoresis (SDS-PAGE) of 50 µl of the resulting eluate was run using a 12% acrylamide Ready Gel™ (Bio-Rad), and the proteins transferred electrophoretically from the gel onto a polyvinylidene diflouride (PVDF) membrane (Millipore). The membrane was blocked for 1 h at room temperature with 1% skimmed milk powder in Tris-buffered saline (TBS), and after washing, the membrane was incubated for 1 h at room temperature with 20 ml of rabbit anti-human uPAR at a concentration of 5 µg $ml^{-1}$. After washing again, treatment continued for 1 h at 23°C with 20 ml of a 1:1000 dilution of murine monoclonal antibody to rabbit immunoglobulins conjugated with alkaline phosphatase. Finally the membrane was washed extensively and phosphatase substrate solution (NBT/BCIP, Sigma) was added to develop colour.

For Western blotting of 48 h conditioned culture medium from MDA-MB-231 BAG cells, the protein-A pre-absorption column was omitted, and 20 ml of the medium was applied directly to the R2 column or the anti-TNP column.

For tumour cell lysates and tumour tissue extracts, 25 µl of the lysate was diluted 1:2 with Laemmli buffer and applied directly to the running gel.

© 1999 Cancer Research Campaign

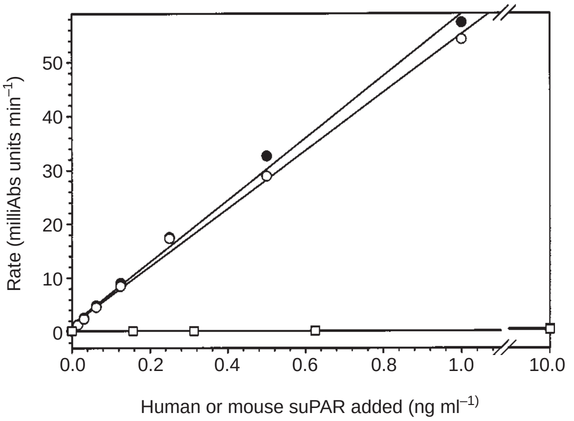

**Figure 1** Recovery of ELISA signal from recombinant human suPAR added in increasing concentrations to assay dilution buffer (●), or a 1:10 dilution of a mouse citrate plasma pool (○). Recovery is also shown for ELISA signal from recombinant mouse suPAR added in increasing concentrations to either assay dilution buffer (■), or to a 1:10 dilution of a mouse citrate plasma pool (□). Values shown are the means of triplicates. The linear correlation coefficient for each fitted line was >0.99

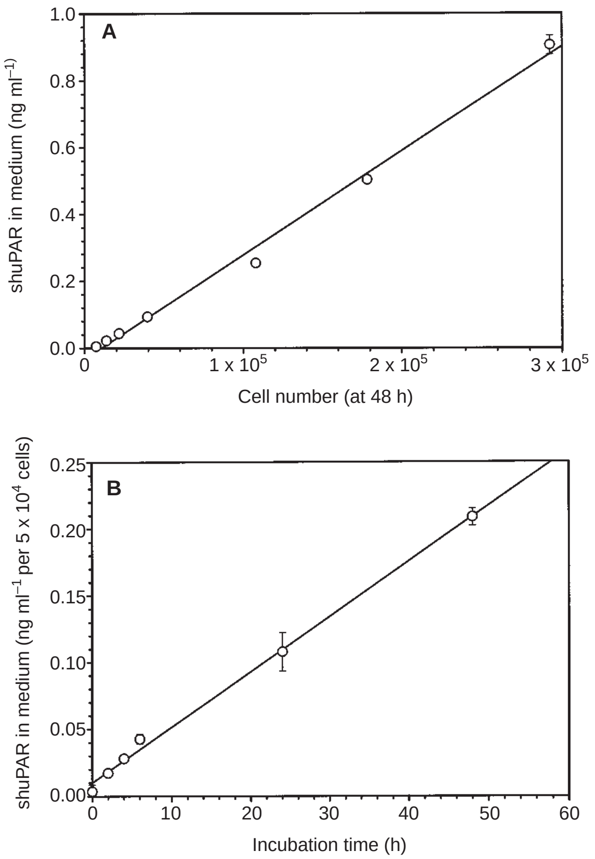

**Figure 2** (**A**) Release of shuPAR from MDA-MB-231 BAG breast cancer cells in vitro. Selected numbers of MDA-MB-231 BAG cells were cultured for 48 h with 1 ml of serum-free medium (SFM). Aliquots of culture supernatants were used for the ELISA of shuPAR. At the end of the experiment the cell number was determined by the MTT-method. Results are expressed as the mean ± s.d. of three independent shuPAR determinations. The linear correlation coefficient for the fitted line was >0.99. (**B**) Release of shuPAR from MDA-MB-231 BAG breast cancer cells in vitro. MDA-MB-231 BAG cells ($2.5 \times 10^4$ cells per well) were cultured for various periods of time with 1 ml of SFM. Aliquots of culture supernatants were tested with the ELISA for shuPAR. At the end of the experiment the cell number was determined by the MTT-method, and the content of shuPAR in the culture supernatants was normalized to $5 \times 10^4$ cells. Results are expressed as the mean ± s.d. of three independent determinations. The linear correlation coefficient for the fitted line was >0.99

### RESULTS

### Validation of shuPAR ELISA

The shuPAR ELISA method employed was capable of accurately measuring recombinant shuPAR over a range of 3 pg ml$^{-1}$ to more than 1 ng ml$^{-1}$. When this standard was added into a 1:10 dilution of mouse citrate plasma the subsequent recovery of signal was better than 90% (Figure 1). The sensitivity to recombinant shuPAR was more than 1000-fold higher than towards recombinant mouse suPAR (Figure 1).

Specificity of the ELISA was rigorously controlled by immunoabsorption experiments as described (Stephens et al, 1997). When conditioned medium (48 h) from MDA-MB-231 BAG cells was absorbed on a protein A-Sepharose column using an anti-uPAR monoclonal antibody (R4) against human uPAR domain 3, different to the R2 antibody used in the ELISA, the shuPAR signal in subsequent ELISA was reduced by approximately 95%.

Using the shuPAR ELISA, no signal above background was obtained from the plasma of nude mice, unless they were host to xenografted human tumours. After 8 weeks growth of subcutaneously implanted MDA-MB-231 BAG tumours, citrate plasma from the host mice gave a substantial shuPAR signal (see below). When this plasma pool was absorbed on a protein A-Sepharose column using the mAb (R4), the signal in subsequent suPAR ELISA was reduced by more than 95%. Thus xenografted human tumours released detectable amounts of shuPAR into the plasma of host mice.

### Measurements of shuPAR released from breast tumour cell lines shuPAR was measured by ELISA in conditioned serum-free media (SFM) from MDA-MB-231 BAG and MDA-MB-435 BAG cells after 24 h of incubation. The concentration of shuPAR in SFM from MDA-MB-231 BAG cells was 0.095 ±0.013 ng ml$^{-1}$ corresponding to 2.2 ±0.14 fg ml$^{-1}$ produced per cell per 24 h (calculated on the basis of the cell number at 24 h). In MDA-MB-435 BAG cultures, the concentration of shuPAR in 48 h conditioned medium was below the detection limit of the ELISA.

To test the relationship between cell number and the shuPAR released into culture supernatant, different numbers of MDA-MB-231 BAG cells were grown in SFM for 48 h, so that there were $10^4$ to $3 \times 10^5$ viable cells per well at the end of the experiment as determined by MTT assay. When shuPAR was measured by ELISA in the conditioned media, it was found that over the range of cell density used there was a close correlation between cell number and shuPAR concentration reached (linear correlation coefficient $r > 0.99$, $P < 0.001$, Figure 2A).

To establish whether shuPAR was continuously released by cells into culture medium as a function of time, conditioned SFM from MDA-MB-231 BAG cells was collected at different time points between 2 and 48 h. For each time point the viable cell number was estimated by the MTT method, and shuPAR levels were determined by ELISA and normalized to $5 \times 10^4$ cells per well. Over this time

 © 1999 Cancer Research Campaign

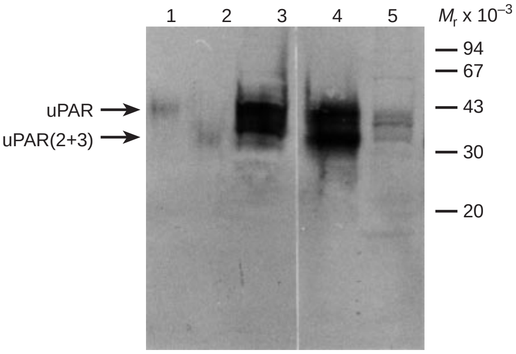

**Figure 3** Western blot analysis of cell extracts of MDA-MB-231 BAG cells. Twenty-five microlitres of total lysate (lane 3), detergent phase (lane 4) and water phase (lane 5) of Triton X-114 extracts (see text) of MDA-MB-231 BAG cells were analysed by Western blotting using polyclonal rabbit anti-human uPAR antibody. The standards consisted of purified full-length shuPAR (lane 1, ~ 100 pg), and chymotrypsin cleaved shuPAR domains (2+3), (lane 2, ~ 200 pg). The arrows indicate the position of full-length shuPAR and shuPAR domains (2+3). Electrophoretic mobility of standard proteins is indicated to the right

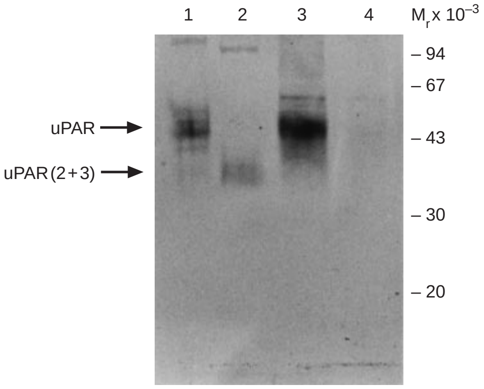

**Figure 4** Western blot analysis of culture supernatants of MDA-MB-231 BAG cells. Forty-eight hours conditioned medium was immunoabsorbed on either an anti-uPAR-R2 column (lane 3) or an anti-TNP column (lane 4), and then analysed together with standards of purified full-length shuPAR (lane 1, ~ 100 pg), and chymotrypsin-cleaved shuPAR domains (2+3), (lane 2, ~ 200 pg), by Western blotting using polyclonal rabbit anti-human uPAR antibodies course the shuPAR level correlated closely with time (linear correlation coefficient $r > 0.99$, $P < 0.001$, Figure 2B).

### Characterization of uPAR in cell lysates and conditioned medium

Cell lysates were made of cultured MDA-MB-231 BAG cells using a Triton X-114 containing buffer together with the protease inhibitors aprotinin, PMSF and EDTA, and then subjected to temperature-induced phase separation (Solberg et al, 1994). Western blotting of the total lysate, detergent phase and water phase of Triton X-114 extracts (without immunoabsorption) showed in all cases two bands corresponding to full-length uPAR and a form consisting of uPAR domains (2+3) (Figure 3), i.e. a cleavage product. No band corresponding to free domain 1 was found in the Western blots of any of these fractions.

Using ultracentrifugation, we then tested whether shuPAR was actually released in soluble form from the cells into the medium, or if vesiculation or blebbing of sedimentable microparticles occurred from the plasma membrane. Medium conditioned by MDA-MB-231 BAG cells for 24 h was either centrifuged at 10 000 ***g*** for 5 min at 4°C or centrifuged at 105 000 ***g*** for 60 min at 4°C, and the concentration of shuPAR determined by ELISA in the resulting supernatants. There was no significant reduction in the concentration of shuPAR measured in the supernatants after either centrifugation (data not shown), suggesting that shuPAR was not present in vesicles or bound to membrane particles. This is in contrast to the findings on sedimentable membrane particles reported to occur in culture supernatants from HT1080 cells (Ginestra et al, 1997).

In order to characterize the molecular form(s) of shuPAR measured in conditioned medium, the R2 monoclonal antibody to human uPAR domain 3 was used for immunoabsorption, and Western blotting was performed on the absorbed shuPAR. In contrast to the mixture of cleaved and uncleaved uPAR found in cell lysates (see above), only full-length three-domain shuPAR was found in the medium from MDA-MB-231 BAG cells (Figure 4). Immunoabsorption and Western blotting was not performed on conditioned medium or cell lysates from MDA-MB-435 BAG cells due to the low shuPAR content.

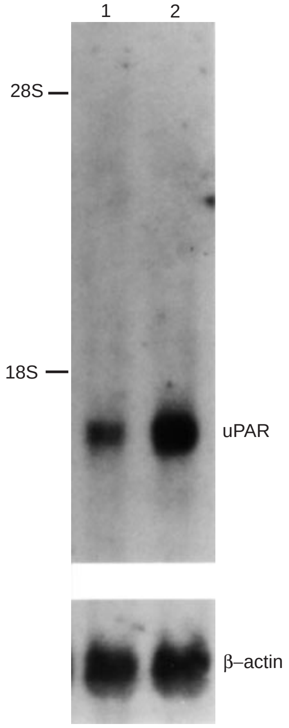

**Figure 5** Northern blot analysis for human uPAR mRNA of extracts of MDA-MB-231 BAG and MDA-MB-435 BAG tumour xenografts. Total RNA was electrophoresed in agarose gels under denaturating conditions and blotted onto a nitrocellulose membrane. The membrane was hybridized with a human cDNA probe for uPAR. Lane 1 contained RNA from MDA-MB-435 BAG tumours and lane 2 contained RNA from MDA-MB-231 BAG tumours. The lanes were loaded with 15 µg of total RNA. The lower section shows a control rehybridization of the membrane with a β-actin cDNA probe. The electrophoretic mobility of ribosomal RNAs is indicated to the left

### Characterization of uPAR in extracts of human tumour xenografts

MDA-MB-231 BAG and MDA-MB-435 BAG tumours grown in nude mice were extracted and analysed by Northern and Western blotting, as well as by ELISA for human uPAR. Northern blot

*© 1999 Cancer Research Campaign*

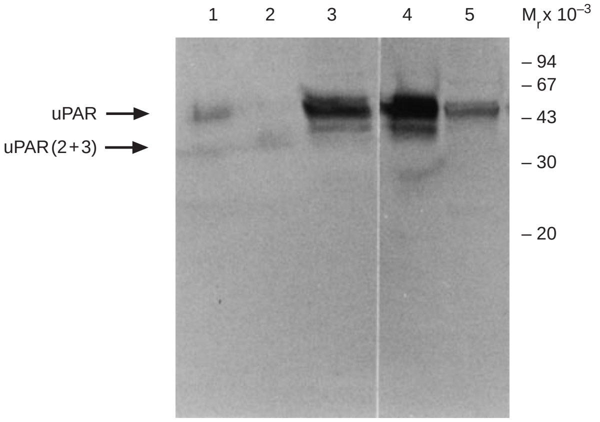

**Figure 6** Western blot analysis of extracts of MDA-MB-231 BAG tumour xenografts. Twenty-five microlitres of total lysate (lane 3), detergent phase (lane 4) or water phase (lane 5) of Triton X-114 extracts (see text) of MDA-MB-231 BAG tumours were analysed by Western blotting using polyclonal rabbit anti-human uPAR antibodies. The standards consisted of purified full-length shuPAR (lane 1, ~ 100 pg), and chymotrypsin-cleaved shuPAR domains (2+3), (lane 2, ~ 200 pg)

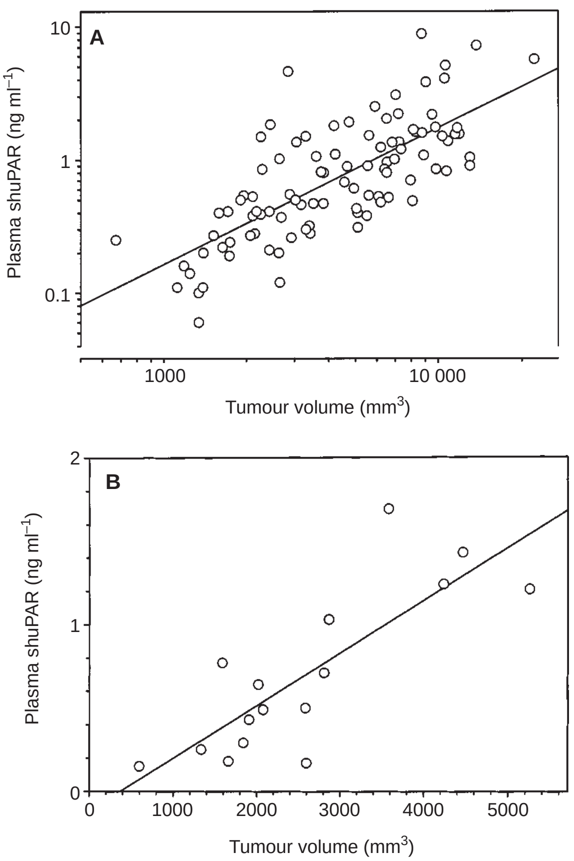

**Figure 7** Comparison between tumour volume and plasma shuPAR level. (**A**) The content of shuPAR in individual host plasma samples from mice xenografted with MDA-MB-231 BAG tumour cells was plotted against the total tumour volume of the tumours on the left and the right flanks of each mouse. The linear correlation coefficient was $r = 0.74$, $P < 0.0001$. (**B**) The content of shuPAR in individual host plasma samples from mice xenografted with HCT 116 tumour cells was plotted against the total tumour volume of the tumours on the left and the right flanks of each mouse. The linear correlation coefficient was $r = 0.81$, $P < 0.0001$

analysis of the xenografted tumours showed that MDA-MB-231 BAG tumours had considerably higher uPAR mRNA expression than MDA-MB-435 BAG tumours (Figure 5) and correspondingly, approximately 5- to 6-fold higher uPAR protein levels (1.074 ± 0.339 ng mg$^{-1}$ protein) than MDA-MB-435 BAG tumours (0.185 ± 0.024 ng mg$^{-1}$ protein). Western blot analysis of MDA-MB-231 BAG tumour tissue total extracts and detergent phase extracts showed two bands, the upper band corresponding to full-length uPAR and the lower band corresponding to uPAR domains (2+3), but only one band (full-length uPAR) was found in the water phase of Triton X-114 extracts. Note that the extraction buffer used included the protease inhibitors aprotinin, PMSF and EDTA (Figure 6). The mobility of the uPAR bands from the tumour extracts did not exactly correspond to the mobility of standard uPAR, probably due to differences in glycosylation (Rønne et al, 1994). A similar Western blot was performed using extracts of MDA-MB-435 BAG tumours, which also showed both full-length uPAR and uPAR domains (2+3) in the total extracts (data not shown). However in these Western blots apparently neither of the two tumour extracts contained free domain 1, as would have been expected from the proteolytic cleavage of uPAR.

### Measurements of shuPAR in plasma from mice with human tumours: relationship between tumour burden and plasma shuPAR

Mice carrying either MDA-MB-231 BAG or MDA-MB-435 BAG tumours had significant levels of shuPAR in their plasma. At a mean tumour volume of 1 cm$^3$, mice carrying MDA-MB-231 BAG tumours had 0.20 ng shuPAR ml$^{-1}$ while mice carrying a 1 cm$^3$ MDA-MB-435 BAG tumour had 3.4 ng shuPAR ml$^{-1}$ in plasma. To study the relationship between released shuPAR and tumour burden, 97 mice were inoculated subcutaneously and bilaterally with MDA-MB-231 BAG tumour cells. Tumours were allowed to grow for 6 weeks ($n = 24$ mice) or 8 weeks ($n = 73$ mice). At these two time points tumour volume was measured and blood was taken for citrate plasma by cardiac puncture. The combined tumour volume for the tumours on the left and right flanks differed for the individual mice in each group, representing the following intervals: 670 mm$^3$ to 7340 mm$^3$ (6 weeks of tumour growth) and 1340 mm$^3$ to 22 200 mm$^3$ (8 weeks of tumour growth). The plasma samples from individual mice were measured by ELISA for shuPAR content. The mean level of plasma shuPAR in the two groups was as follows: 0.54 ± 0.41 ng ml$^{-1}$ (6 weeks of tumour growth); 1.23 ± 1.3 ng ml$^{-1}$ (8 weeks of tumour growth). When the shuPAR levels were plotted against the corresponding total tumour volume for each individual mouse, it was found that plasma shuPAR correlated significantly with tumour volume (linear correlation coefficient $r = 0.74$, $P < 0.0001$, Figure 7A).

In order to test whether the correlation between plasma shuPAR and tumour volume could be observed for another type of cancer xenograft, the human colon tumour cell line, HCT 116, was inoculated subcutaneously and bilaterally into 16 *nu/nu*–META/Bom mice. Tumours were allowed to grow for 5 weeks ($n = 9$ mice) or 8 weeks ($n = 7$ mice). The combined tumour volume for the tumours on the left and right flanks differed for the individual mice in each group, representing the following intervals: 1340 mm$^3$ to 4470 mm$^3$ (5 weeks of tumour growth) and 600 mm$^3$ to 5260 mm$^3$ (8 weeks of tumour growth). The mean tumour volume between the two groups was not significantly different. The plasma samples from individual mice were measured by ELISA for shuPAR

*© 1999 Cancer Research Campaign*

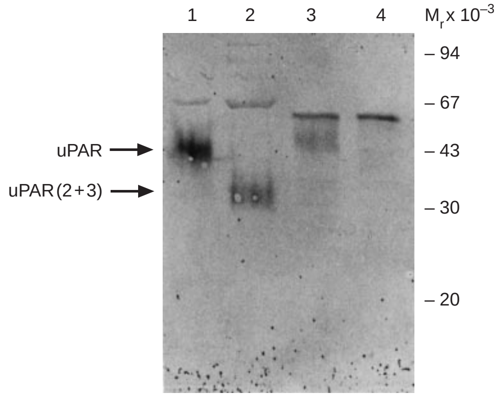

**Figure 8** Western blot analysis of citrate plasma from mice xenografted with MDA-MB-231 BAG tumours. A pool of citrate plasma from mice with MDA-MB-231 BAG tumours was immunoabsorbed on either an anti-uPAR-R2 column (lane 3) or an anti-TNP column (lane 4), and then analysed together with purified full-length shuPAR (lane 1, ~ 100 pg), and cleaved shuPAR domains (2+3), (lane 2, ~ 200 pg), by Western blotting using polyclonal rabbit anti-human uPAR antibodies content. The mean level of plasma shuPAR in the two groups was as follows: 0.73 ± 0.55 ng $ml^{-1}$ (5 weeks of tumour growth); 0.66 ± 0.44 ng $ml^{-1}$ (8 weeks of tumour growth). When the plasma shuPAR levels were plotted against the corresponding total tumour volume for each individual mouse, it was found that plasma shuPAR correlated closely with tumour volume (linear correlation coefficient $r = 0.81$, $P < 0.0001$, Figure 7B). The amount of uPAR protein in HCT 116 tumour extracts was 0.728 ± 0.123 ng $mg^{-1}$ protein, and Western blot analysis of the tumour tissue extracts showed two bands, the upper band corresponding to full-length uPAR and the lower band corresponding to uPAR domains (2+3) (data not shown).

### Characterization of shuPAR in plasma from mice with human tumours

To characterize the molecular form(s) of shuPAR measured in the plasma of mice bearing human tumour xenografts, plasma was immunoabsorbed with the R2 monoclonal antibody to uPAR, and the immunoconcentrated shuPAR was then examined by Western blotting. Plasma from mice with MDA-MB-231 BAG tumours showed only one band corresponding to full-length shuPAR, and no shuPAR was found in the form of domains (2+3) (Figure 8). A similar Western blot was performed using plasma from mice with MDA-MB-435 BAG and HCT 116 tumours, which also showed only one band corresponding to full-length shuPAR (data not shown).

### DISCUSSION

This study was facilitated by the use of a newly developed shuPAR ELISA which achieves a high level of sensitivity while maintaining strict specificity for human uPAR. The assay was tested in several different ways in both purified systems as well as with internal controls of recombinant shuPAR and smuPAR added into a pool of mouse plasma. Specificity was also rigorously controlled by immunoabsorption experiments. Since the assay was over 1000 times more sensitive to huPAR than muPAR, and signal was only found at significant levels in plasma from mice bearing human tumours, it was concluded that the suPAR signal measured in plasma from tumour-bearing mice represented shuPAR.

By applying the shuPAR ELISA to conditioned media from MDA-MB-231 BAG human breast cancer cells, it was confirmed that these cells released shuPAR into the medium. The release of shuPAR was both time-and cell number-dependent, suggesting a constant and direct correlation between cell number and amount of shuPAR released from the cells. Furthermore, when the two human breast cancer cell lines were grown as solid tumours in nude mice, shuPAR was released from the tumour cells into the blood. A highly significant correlation was found between tumour volume and plasma shuPAR concentration in MDA-MB-231 BAG and HCT 116 xenografted mice, indicating that plasma levels of shuPAR reflect tumour burden in the host mice.

The close correlation between plasma shuPAR content and tumour volume suggests that shuPAR may be useful in monitoring increasing or decreasing tumour burden. We have recently found that patients with metastatic breast, lung and colon cancer have increased levels of shuPAR in their blood (Pappot et al, 1997; Stephens et al, 1997), and that plasma suPAR levels correlate with stage of disease and patient survival in colorectal and ovarian cancer patients (Stephens et al, 1997; Sier et al, 1998). It remains to be demonstrated, however, both in the experimental mouse model and in cancer patients, whether plasma levels of shuPAR also will reflect tumour regression, when growth is impaired by an anti-tumour agent such as a cytotoxic drug, i.e. whether shuPAR can be used as a surrogate end point in the treatment of cancer patients.

The mAb R2 used in the ELISA is directed against an epitope located in domain 3 of uPAR. Thus, the assay will detect both full-length uPAR and the form consisting of domains (2+3). In order to further characterize the form(s) of shuPAR measured in cell culture supernatants, Western blot experiments were performed. It was found that in conditioned medium of MDA-MB-231 BAG cells, only the full-length form of shuPAR was released from the tumour cells and not the domains (2+3) form seen in cell lysates. This suggests that either a phospholipase cleavage of the GPI-anchor or a proteolytic cleavage at the carboxy-terminal end of uPAR could have taken place. Since shuPAR was released under serum-free conditions, it seems that the underlying mechanism(s) involves cell-associated processes.

The study was then extended to investigate the form(s) of shuPAR measured in plasma from the mice bearing the human tumours. The MDA-MB-231 BAG human cancer cells were injected into nude mice and allowed to form solid tumours. Western blotting of immunoconcentrated plasma from mice with MDA-MB-231 BAG tumours showed the presence of full-length shuPAR in vivo, which is in agreement with the findings in conditioned medium and in patients with different forms of cancer (Stephens et al, manuscript in preparation). Similar results were obtained in plasma from both MDA-MB-435 BAG and HCT 116 tumour-bearing animals. Thus, despite the presence of domains (2+3) in cell and tumour extracts, only the full-length receptor was detected in conditioned media and plasma. However, due to the detection limit of the Western blotting, it cannot be completely excluded that a minor fraction of domains (2+3) and domain 1 uPAR is present in conditioned medium and/or plasma. One possible explanation for this differential release could be that domains (2+3) uPAR is somehow protected against further enzymatic cleavage which would release it from the cell surface.

In human breast and colon carcinomas, uPAR is expressed by tumour-infiltrating macrophages in addition to the cancer cells (Pyke et al, 1991, 1993). In our breast and colon cancer xenograft

© 1999 Cancer Research Campaign 

models, the measured suPAR is clearly derived from the human cancer cells, so this may only partially represent the situation for release of suPAR in cancer patients. However, our models may be useful in studying the mechanism by which suPAR is released from tumour cells in vivo.

One possible biological role of shuPAR could be as a scavenger for uPA, thus reducing the proportion of uPA bound to cell-surface uPAR (Masucci et al, 1991). It has been shown in vitro that relatively high concentrations of suPAR can act as a scavenger for uPA and thereby inhibit uPA mediated proliferation and Matrigel invasion of human tumour cells (Wilhelm et al, 1994). However, this role is not thermodynamically favoured in vivo, since the physiological concentrations of both uPA and uPAR in plasma (approximately 20 pM) are well below that required for a significant level of interaction ($K_d$ = 0.1 nM). At the pericellular level, however, local shuPAR levels may reach levels that could attenuate uPA dependent tumour cell behaviour. Another putative role of suPAR could be as a chemotactic stimulator, although this activity is specifically related to the chymotrypsin-cleaved suPAR, i.e. domains (2+3) uPAR (Resnati et al, 1996). In addition, suPAR could contribute to enhanced detachment of tumour cells from the surrounding extracellular matrix by competing with cell-surface uPAR for vitronectin binding (Høyer-Hansen et al, 1997; Chavakis et al, 1998).

### ACKNOWLEDGEMENTS

This study was kindly supported by Else and Mogens Wedell-Wedelsborg' Foundation, The Foundation of 17.12.1981 and The Danish Cancer Society.

### REFERENCES

Andreasen PA, Kjøller L, Christensen L and Duffy MJ (1997) The urokinase-type plasminogen activator system in cancer metastasis: a review. *Int J Cancer* **72**: 1–22

Baramova EN, Bajou K, Remacle A, Lhoir C, Krell HW, Weidle UH, Nöel A and Foidart JM (1997) Involvement of PA/plasmin system in the processing of pro-MMP-9 and in the second step of pro-MMP-2 activation. *FEBS Lett* **405**: 157–162

Behrendt N, Rønne E, Ploug M, Petri T, Løber D, Nielsen LS, Schleuning WD, Blasi F, Appella E and Danø K (1990) The human receptor for urokinase plasminogen activator. NH2-terminal amino acid sequence and glycosylation variants. *J Biol Chem* **265**: 6453–6460

Brünner N, Thompson EW, Spang-Thomsen M, Rygaard J, Danø K and Zwiebel JA (1992) lacZ transduced human breast cancer xenografts as an in vivo model for the study of invasion and metastasis. *Eur J Cancer* **28A**: 1989–1995

Chavakis T, Kanse SM, Yutzy B, Lijnen HR and Preissner KT (1998) Vitronectin concentrates proteolytic activity on the cell surface and extracellular matrix by trapping soluble urokinase receptor–urokinase complexes. *Blood* **91**: 2305–2312

Chomczynski P and Sacchi N (1987) Single-step method of RNA isolation by acid guanidinium thiocyanate-phenol-chloroform extraction. *Anal Biochem* **162**: 156–159

Danø K, Andreasen PA, Grøndahl-Hansen J, Kristensen P, Nielsen LS and Skriver L (1985) Plasminogen activators, tissue degradation, and cancer. *Adv Cancer Res* **44**: 139–266

Danø K, Behrendt N, Brünner N, Ellis V, Ploug M and Pyke C (1994) The urokinase receptor. Protein structure and role in plasminogen activation and cancer invasion. *Fibrinolysis* **8**: 189–203

Deng G, Curriden SA, Wang SJ, Rosenberg S and Loskutoff DJ (1996) Is plasminogen activator inhibitor-1 the molecular switch that governs urokinase receptor-mediated cell adhesion and release. *J Cell Biol* **134**: 1563–1571

Ellis V, Behrendt N and Danø K (1991) Plasminogen activation by receptor-bound urokinase. A kinetic study with both cell-associated and isolated receptor. *J Biol Chem* **266**: 12752–12758

Feinberg AP and Vogelstein B (1983) A technique for radiolabeling DNA restriction endonuclease fragments to high specific activity. *Anal Biochem* **132**: 6–13

Ganesh S, Sier CF, Heerding MM, Griffioen G, Lamers CB and Verspaget HW (1994) Urokinase receptor and colorectal cancer survival. *Lancet* **344**: 401–402

Ginestra A, Monea S, Seghezzi G, Dolo V, Nagase H, Mignatti P and Vittorelli ML (1997) Urokinase plasminogen activator and gelatinases are associated with membrane vesicles shed by human HT1080 fibrosarcoma cells. *J Biol Chem* **272**: 17216–17222

Grøndahl-Hansen J, Peters HA, Van PW, Look MP, Pappot H, Rønne E, Danø K, Klijn JM, Brünner N and Foekens JA (1995) Prognostic significance of the receptor for urokinase plasminogen activator in breast cancer. *Clin Can Res* 1079–1087

He CS, Wilhelm SM, Pentland AP, Marmer BL, Grant GA, Eisen AZ and Goldberg GI (1989) Tissue cooperation in a proteolytic cascade activating human interstitial collagenase. *Proc Natl Acad Sci USA* **86**: 2632–2636

Holst-Hansen C, Johannessen B, Høyer-Hansen G, Rømer J, Ellis V and Brünner N (1996) Urokinase-type plasminogen activation in three human breast cancer cell lines correlates with their in vitro invasiveness. *Clin Exp Metastasis* **14**: 297–307

Høyer-Hansen G, Behrendt N, Ploug M, Danø K and Preissner KT (1997) The intact urokinase receptor is required for efficient vitronectin binding: receptor cleavage prevents ligand interaction. *FEBS Lett* **420**: 79–85

Lau HK and Kim M (1994) Soluble urokinase receptor from fibrosarcoma HT-1080 cells. *Blood Coagul Fibrinolysis* **5**: 473–478

Lyons RM, Gentry LE, Purchio AF and Moses HL (1990) Mechanism of activation of latent recombinant transforming growth factor beta 1 by plasmin. *J Cell Biol* **110**: 1361–1367

Masucci MT, Pedersen N and Blasi F (1991) A soluble, ligand binding mutant of the human urokinase plasminogen activator receptor. *J Biol Chem* **266**: 8655–8658

Pappot H, Høyer-Hansen G, Rønne E, Hansen HH, Brünner N, Danø K and Grøndahl-Hansen J (1997) Elevated plasma levels of urokinase plasminogen activator receptor in non-small cell lung cancer patients. *Eur J Cancer* **33**: 867–872

Pedersen N, Schmitt M, Rønne E, Nicoletti MI, Høyer-Hansen G, Conese M, Giavazzi R, Danø K, Kuhn W, Jänicke F and Blasi F (1993) A ligand-free, soluble urokinase receptor is present in the ascitic fluid from patients with ovarian cancer. *J Clin Invest* **92**: 2160–2167

Pedersen H, Brünner N, Francis D, Østerlind K, Rønne E, Hansen HH, Danø K and Grøndahl-Hansen J (1994) Prognostic impact of urokinase, urokinase receptor, and type 1 plasminogen activator inhibitor in squamous and large cell lung cancer tissue. *Cancer Res* **54**: 4671–4675

Plesner T, Behrendt N and Ploug M (1997) Structure, function and expression on blood and bone marrow cells of the urokinase-type plasminogen activator receptor, uPAR. *Stem Cells* **15**: 398–408

Ploug M, Rønne E, Behrendt N, Jensen AL, Blasi F and Danø K (1991) Cellular receptor for urokinase plasminogen activator. Carboxyl-terminal processing and membrane anchoring by glycosyl-phosphatidylinositol. *J Biol Chem* **266**: 1926–1933

Ploug M, Ellis V and Danø K (1994) Ligand interaction between urokinase-type plasminogen activator and its receptor probed with 8-anilino-1-naphthalenesulfonate. Evidence for a hydrophobic binding site exposed only on the intact receptor. *Biochemistry* **33**: 8991–8997

Ploug M, Østergaard S, Hansen LBL, Holm A and Danø K (1998) Photoaffinity labeling of the human receptor for urokinase-type plasminogen activator using a decapeptide antagonist. Evidence for a composite ligand-binding site and a short interdomain separation. *Biochemistry* **37**: 3612–3622

Ponte P, Ng SY, Engel J, Gunning P and Kedes L (1984) Evolutionary conservation in the untranslated regions of actin mRNAs: DNA sequence of a human beta-actin cDNA. *Nucleic Acids Res* **12**: 1687–1696

Pyke C, Kristensen P, Ralfkiaer E, Grøndahl-Hansen J, Eriksen J, Blasi F and Danø K (1991) Urokinase-type plasminogen activator is expressed in stromal cells and its receptor in cancer cells at invasive foci in human colon adenocarcinomas. *Am J Pathol* **138**: 1059–1067

Pyke C, Graem N, Ralfkiaer E, Rømer J, Høyer-Hansen G, Brünner N and Danø K (1993) Receptor for urokinase is present in tumor-associated macrophages in ductal breast carcinoma. *Cancer Res* **15**: 1911–1915

Resnati M, Guttinger M, Valcamonica S, Sidenius N, Blasi F and Fazioli F (1996) Proteolytic cleavage of the urokinase receptor substitutes for the agonist-induced chemotactic effect. *EMBO J* **15**: 1572–1582

Roldan AL, Cubellis MV, Masucci MT, Behrendt N, Lund LR, Danø K, Appella E and Blasi F (1990) Cloning and expression of the receptor for human urokinase plasminogen activator, a central molecule in cell surface, plasmin dependent proteolysis. *EMBO J* **9**: 467–474

 © 1999 Cancer Research Campaign

Rømer J, Pyke C, Lund LR, Eriksen J, Kristensen P, Rønne E, Høyer-Hansen G, Danø K and Brünner N (1994) Expression of uPA and its receptor by both neoplastic and stromal cells during xenograft invasion. *Int J Cancer* **57**: 553–560

Rønne E, Behrendt N, Ploug M, Nielsen HJ, Wollisch E, Weidle U, Danø K and Høyer-Hansen G (1994) Quantitation of the receptor for urokinase plasminogen activator by enzyme-linked linked immunosorbent assay. *J Immunol Methods* **167**: 91–101

Rønne E, Høyer-Hansen G, Brünner N, Pedersen H, Rank F, Osborne CK, Clark GM, Danø K and Grøndahl-Hansen J (1995*a*) Urokinase receptor in breast cancer tissue extracts. Enzyme-linked immunosorbent assay with a combination of mono- and polyclonal antibodies. *Breast Cancer Res Treat* **33**: 199–207

Rønne E, Pappot H, Grøndahl-Hansen J, Høyer-Hansen G, Plesner T, Hansen NE and Danø K (1995*b*) The receptor for urokinase plasminogen activator is present in plasma from healthy donors and elevated in patients with paroxysmal nocturnal haemoglobinuria. *Br J Haematol* **89**: 576–581

Shapiro RL, Duquette JG, Roses DF, Nunes I, Harris MN, Kamino H, Wilson EL and Rifkin DB (1996) Induction of primary cutaneous melanocytic neoplasms in urokinase-type plasminogen activator (uPA)-deficient and wild-type mice – cellular blue nevi invade but do not progress to malignant melanoma in uPA-deficient animals. *Cancer Res* **56**: 3597–3604

Sier CFM, Stephens RW, Bizik J, Mariani A, Bassan M, Pedersen N, Frigerio L, Ferrari A, Danø K, Brünner N and Blasi F (1998) The level of urokinase plasminogen activator receptor (uPAR) is increased in serum of ovarian cancer patients. *Cancer Res* **58**: 1843–1849

Solberg H, Rømer J, Brünner N, Holm A, Sidenius N, Danø K and Høyer-Hansen G (1994) A cleaved form of the receptor for urokinase-type plasminogen activator in invasive transplanted human and murine tumors. *Int J Cancer* **58**: 877–881

Stephens RW, Pedersen AN, Nielsen HJ, Hamers MJ, Høyer-Hansen G, Rønne E, Dybkjær E, Danø K and Brünner N (1997) ELISA determination of soluble urokinase receptor in blood from healthy donors and cancer patients. *Clin Chem* **43**: 1868–1876

Stephens RW, Nielsen HJ, Christensen IJ, Thorlacius-Ussing O, Sørensen S, Danø K and Brünner N (1999) Plasma urokinase receptor levels in patients with colorectal cancer: relationship to prognosis. *J Natl Cancer Inst* **91**: 869–874

Wilhelm O, Weidle U, Hohl S, Rettenberger P, Schmitt M and Graeff H (1994) Recombinant soluble urokinase receptor as a scavenger for urokinase-type plasminogen activator (uPA). Inhibition of proliferation and invasion of human ovarian cancer cells. *FEBS Lett* **337**: 131–134

Wohn KD, Kanse SM, Deutsch V, Schmidt T, Eldor A and Preissner KT (1997) The urokinase-receptor (CD87) is expressed in cells of the megakaryoblastic lineage. *Thromb Haemost* **77**: 540–547

*© 1999 Cancer Research Campaign*
